# Supplementary material for: Sandwich (Amnion/Conjunctival-Limbal Autograft/Amnion) Transplantation for Recurrent Pterygium with Restrictive Strabismus
Source: J Clin Med. 2022 Dec 3;11(23):7193. doi: 10.3390/jcm11237193 (PMC9737167; doi:10.3390/jcm11237193)
Supplement: Supplementary file 1 [file jcm-11-07193-s001.zip › jcm-2032703-supplementary.pdf]

Table S1. Preoperative and Postoperative Measurements for Patients Undergoing Sandwich Transplantation following Recurrent Pterygium Excision.

Eye Number	Age (Years)	Gender	Number of Prior Surgeries	Best-Corrected Visual Acuity		Symble-pharon	Primary position		Fornix Reconstruction	Follow-up (months)	Other Diagnosis
				Pre-op	Post-op		Pre-op Esotropia (PD)	Post-op			
1.	46	M	3 OS	1.0	1.0	+	25	Orthotropia	+	12	No
2.	36	F	4 OD	1.0	1.0	+	20	Orthotropia	+	36	No
3.	80	M	1 OS	0.1	0.3	+	20	Orthotropia	+	32	Cataract
4.	60	F	3 OS	CF/15cm	0.6	+	30	Orthotropia	+	31	Cataract
5.	60	M	1 OD	0.3	0.5	-	15	Orthotropia	-	12	Cataract
6.	68	M	1 OS	0.6	0.6	-	10	Orthotropia	-	18	Cataract
7.	55	M	1 OS	1.0	1.0	+	15	Orthotropia	+	20	No
8.	54	F	2 OS	0.1	0.3	-	17	Orthotropia	-	14	Cataract
9.	66	M	1 OS	0.4	0.4	-	12	Orthotropia	-	15	Cataract
10.	73	F	1 OS	0.8	0.8	-	16	Orthotropia	-	16	IOL insertion
11.	68	F	2 OS	0.1	0.4	-	10	Orthotropia	-	13	Cataract

** Abbreviations: F= Female; M= Male; OD= Right eye; OS= Left eye; - = No; + = Yes.*
